# Impact of the Timing and Temperature of Malolactic Fermentation on the Aroma Composition and Mouthfeel Properties of Chardonnay Wine

**DOI:** 10.3390/foods9060802

**Published:** 2020-06-18

**Authors:** Anthony Sereni, Quynh Phan, James Osborne, Elizabeth Tomasino

**Affiliations:** Department of Food Science & Technology, Oregon State University, Corvallis, OR 97331, USA; anthony.sereni@oregonstate.edu (A.S.); quynh.phan@oregonstate.edu (Q.P.); james.osborne@oregonstate.edu (J.O.)

**Keywords:** *Saccharomyces cerevisiae*, *Oenococcus oeni*, *Torulaspora delbrueckii*, concurrent fermentation, GCMS, Napping^®^, ultra-flash profiling

## Abstract

Malolactic fermentation (MLF) is an important process in wine production due to the resulting reduction in acidity. MLF is typically induced by the addition of *Oenococcus oeni* after the completion of alcoholic fermentation (AF), but can occur concurrent with AF by co-inoculation of *O. oeni* with *Saccharomyces cerevisiae*. This study investigated the effect of MLF inoculation timing and temperature (15 °C and 21 °C) and the presence of the non-*Saccharomyces* yeast *Torulaspora delbrueckii* on Chardonnay wine aroma and mouthfeel. Aroma composition was measured using headspace solid-phase microextraction-gas chromatography mass spectrometry (HS-SPME-GCMS). Mouthfeel attributes of the wines produced were assessed by a winemaker panel, using Napping^®^ and Ultra-flash profiling. Significant differences in aroma composition and mouthfeel perception were found based on MLF timing and inoculation conditions, as well as between temperatures. Temperature had a greater impact on the aroma composition for sequential inoculations, while there were little differences based on the temperature of concurrent fermentations. Treatment type and temperature also affected the chemical composition of finished wines. Mouthfeel was impacted, although not as strongly as aroma composition. These findings demonstrate the usefulness of various MLF practices to influence the sensory qualities of a Chardonnay wine.

## 1. Introduction

Chardonnay is referred to as a neutral aromatic grape cultivar, producing a wine that is not defined by a specific set of aroma compounds [1,2]. Chardonnay wine is possibly the most diverse white wine style, allowing for many variations in processing steps, including a variety of styles in sparkling wine and some dessert wines. However, it is most commonly used for the production of still white wine [3]. Chardonnay grapes are typically pressed before alcoholic fermentation (AF) to minimize contact with the skin and seeds [1]. In the absence of the aromatic constituents of grape skins, Chardonnay is most often fermented at one of two temperature ranges to retain aroma compounds; 10–16 °C or 20–25 °C [4]. However, the impact of fermentation temperature on mouthfeel has been little studied. Of almost all white wines, texture and mouthfeel are considered of extreme importance for the Chardonnay wine style [5].

Another important winemaking process for many styles of Chardonnay wine is malolactic fermentation (MLF). This process results in a raise of pH in the wine and an increase in microbial stability due to the removal of malic acid (a potential nutrient source for spoilage lactic acid bacteria) [6]. Because of the decrease in acidity, this process is conducted in cooler climate cultivars, such as Chardonnay, which generally have higher acidity than warm climate grapes [7]. MLF is generally conducted after AF, with the addition of *Oenococcus oeni*, for the conversion of diprotic malic acid to lactic acid (single protic group). This process is also known to alter the sensory characteristics of wines. Avedovech et al. [8] found that tasters could discern differences in aroma between Chardonnay wines that have undergone MLF vs. non-MLF treatments. Subsequent volatile composition analysis by Gas Chromatography Mass Spectrometry (GCMS) also showed significant differences. MLF produced a variety of desired aroma compounds, including diacetyl, acetoin, volatile acids, diethyl succinate, volatile esters, ethyl acetate, n-propanol, 2-butanol, n-hexanol, ethyl lactate, and 2,3-butanediol.

While MLF is typically conducted after the completion of the alcoholic fermentation, it may also occur at the same time as the alcoholic fermentation (AF). This is known as either concurrent or simultaneous fermentation and is induced by the inoculation of both the yeast and bacterial starter cultures at the same time. In red winemaking, concurrent inoculation has been studied as a possible means of reliably completing AF and MLF in a shorter period of time. For example, AF and MLF were shown to more reliably complete fermentation with concurrent inoculations than sequential AF and MLF [9]. Concurrent AF and MLF is increasingly being used in modern winemaking, but there are still anecdotal concerns of higher levels of volatile acidity and stuck fermentations. This is due to the fact that *O. oeni* is a heterofermentative bacteria that can produce acetic acid via the metabolism of glucose [10]. However, due to the bacteria’s preference for malic acid metabolism at pH levels <3.60, increased acetic acid has only been noted when concurrent inoculation occurred in high pH juices/musts [11].

A number of studies have reported on the impact of concurrent inoculation on wine aroma [12,13,14,15]. Unfortunately, few have reported on the influence of MLF timing on wine mouthfeel. For example, Maarman et al. [15] found increases in volatile esters including ethyl acetate when using concurrent inoculations versus sequential inoculations. However, sensory analysis was not conducted on these wines, so no comparison of wine mouthfeel could be made. In a study conducted in Shiraz, Abrahamse and Bartowsky [12] reported significant differences in volatile compounds as well as anthocyanin and pigmented polymer composition. Again, no sensory analysis of the wines was conducted, so the influence of MLF timing on wine mouthfeel could not be determined.

If concurrent fermentations are to be performed, the presence of other micro-organisms at the beginning of fermentation must be considered. After grapes are harvested and processed, a large number of yeast and bacteria species may still be present on the grapes. How the presence of these microbes influences the ability of *O. oeni* to conduct a concurrent MLF is relatively unknown. A number of studies have investigated how the presence of microorganisms naturally present on the grapes at harvest impact *Saccharomyces cerevisiae.* Many of these yeast species present on grapes cannot survive the high alcohol environment created by the fermentation by *Saccharomyces cerevisiae*, but can still interfere with the health of *Saccharomyces cerevisiae* by limiting nutrient availability or by the generation of harmful compounds [16,17]. Arnink et al. [18] demonstrated the negative impact of nutrient stress on *S. cerevisiae* and *O. oeni* during AF and MLF, with particular importance on nitrogen availability. High microbial loads can also influence the sensory properties of a finished wine in both positive and negative ways [17].

Some non-*Saccharomyces* yeast species can positively impact wine flavor and aroma when present before or during fermentation with *S. cerevisiae*. In fact, a number of non-*Saccharomyces* yeast are now available as commercial cultures. *Metschnikowia pulcherrima* has been shown to decrease final wine alcohol content from 0.9–1.6%, with positive sensory aspects noted for Shiraz wines, but a negative aromatic influence imparted into Chardonnay wine due to increased levels of ethyl acetate (aroma described as nail polish remover) [19]. *Hanseniaspora vineae,* an apiculate yeast species, has been cited to remain active in ferment until up to 9% alcohol (*v*/*v*) and in Chardonnay wines, it can be associated with an increase in “fruit intensity, described as banana, pear, apple, citric fruits, and guava.” [20]. Englezoset et al. [21] has shown that different yeast species and inoculation protocol greatly impact the growth of lactic acid bacteria and the wines’ aroma composition. Despite the importance of non-*Saccharomyces* yeast to the winemaking practice, little is known about how their presence may impact the MLF. This is of particular importance when considering concurrent fermentations as it is unknown how *O. oeni* will react when inoculated into a grape juice/where a high population of non-*Saccharomyces* may be present.

The objective of this study was, therefore, to investigate how the timing of MLF impacts the aroma and mouthfeel of Chardonnay wine. Two different temperatures of fermentation were explored as Chardonnay is commonly fermented at either a cool (13–15 °C) or warm (18–21 °C) temperature to produce different styles of wine. The impact of a high population of a non-*Saccharomyces* yeast on co-inoculated fermentations was also investigated as the use of this yeast in winemaking is increasing, but little is known about how this may impact MLF.

## 2. Materials and Methods

### 2.1. Winemaking

**Figure 1:**
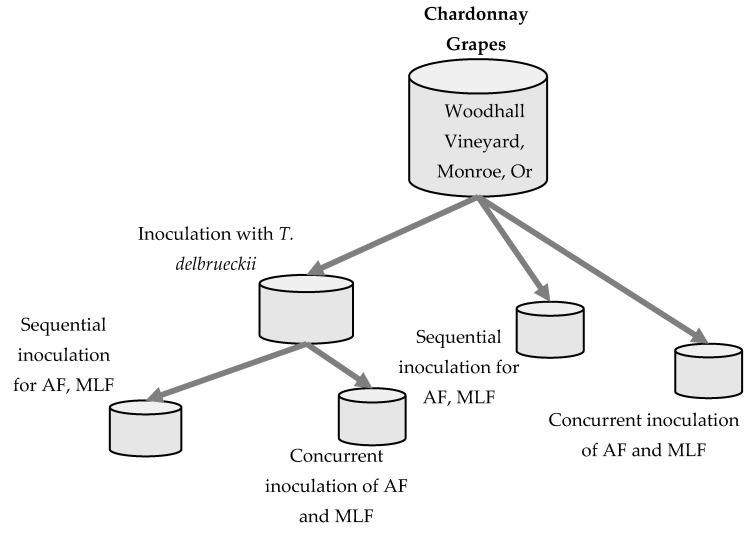
Chardonnay winemaking treatments (AF = alcoholic fermentation with *S. cerevisiae* D47), MLF = malolactic fermentation with *O. oeni* beta). Winemaking treatments were done at 15 °C and 21 °C. AF was conducted by *S. cerevisiae* D47 and MLF by *O. oeni* Beta.

Chardonnay grapes were harvested September 2014 from Oregon State University’s Woodhall vineyard (Monroe, OR, USA). A destemmer (VLS technologies, Treviso, Italy) was used to destem the grapes, which were then pressed at 0.1 MPa for 15 min using a membrane press (Velo technologies, Treviso, Italy). The resulting juice was settled for 12 h at 8 °C. After racking, the juice was divided into 24 one-gallon glass carboys, 3 L per carboy, and secured with airlocks. A commercial culture of *Torulaspora delbrueckii* (Vinoflora Prelude™) (Chr. Hansen, Hørsholm, Denmark) was added to one set of carboys at a rate of 0.25 g/L after hydration according to the manufacturer’s specification and the juice was held at either 15 or 21 °C for 48 h. After 48 h, carboys were inoculated with *Saccharomyces cerevisiae* D47 (Lallemand, Montreal, Canada) at a rate of 0.25 g/L after hydration according to the manufacturer’s specification. Carboys of juice to which *T. delbrueckii* was not added were also inoculated with *S. cerevisiae* D47 after the initial settling step. At the time of *S. cerevisiae* inoculation, half of the carboys were also inoculated with *Oenococcus oeni* Beta (Lallemand) to induce MLF. *O. oeni* was inoculated at approximately 1 × 10^6^ cfu/mL following the manufacturer’s instructions. For the remaining carboys, Beta was inoculated at the completion of alcoholic fermentation. All fermentations were performed in triplicate at either 15 or 21 °C. Figure 1 displays a flow chart of the treatment details.

At the completion of AF and MLF (glucose + fructose <4 g/L, malic acid <50 mg/L), an addition of 50 mg/L SO_2_ was made to the wines before they were placed at 4 °C to settle. After 14 days of settling, the wines were racked and free SO_2_ was adjusted to approximately 25 mg/L. Samples from each replicate were taken and frozen at −20 °C until required for analysis. Replicates were then combined, sterile filtered (0.45 μm PES cartridge filter), and bottled in 375 mL green glass bottles sparged with nitrogen and sealed with aluminum screw cap closures (Stelvin ^TM^, Amcor, Australia).

### 2.2. Chemical Analysis

Basic juice analysis included total soluble solids, pH, and titratable acidity (TA). Soluble solids were monitored throughout AF using a digital densitometer (Anton Paar, Santner Foundation, Graz, Austria). pH was determined by ion-selective electrode (ThermoFisher Scientific, MA, USA) and TA by titration with 0.1 M NaOH. Glucose/fructose, malic acid, and acetic acid were measured by enzymatic test kits (r-Biopharm, Darmstadt, Germany), while ethanol was determined using an Alcolyzer (Anton Paar, Santner Foundation, Graz, Austria).

Aroma compounds were measured using HS-SPME-GCMS, adapting methodology 1 from previously published [22]. Samples were run on a Shimadzu GCMS-QP2010 (Shimadzu Scientific Instruments Inc., Columbia, MD, USA) equipped with a CTC Combi-Pal autosampler fitted with a stack cooler (CTC-Analytics AG, Switzerland). Sample preparation, extraction, fibers, columns, oven ramp, and all other method parameters are the same as Tomasino et al. [22]. Samples were analyzed on a Shimazdu QP2010 GCMS (Shimazdu, Columbia, MD, USA) equipped with a Shimazdu Combi-Pal AOC-5000 plus auto-sampler. A 2 cm long Stablefelx DVB/CAR/PDMS combination SPME fiber (50/30 µm thickness, 24 gauge, Supleco, Bellefonte, PA) was used for HS-SPME. Prior to use, the SPME fiber was conditioned at 250 °C in the injection port for 1 h. Prior to each sample analysis, the SPME fiber was further conditioned in an NDL heater attached to the Combo-Pal autosampler in nitrogen for 10 min at 250 °C.

The chromatography configuration contained dual columns connected in series using a deactivated universal press-tight connector (Restek, Bellefonte, PA, USA); a Stabilwax column (30 m × 0.25 mm ID × 0.5 μm film thickness, polyethylene glycol, Restek) connected to a Rxi-1MS column (15 m × 0.25 mm ID × 0.5 μm film thickness, 100% dimethyl polysiloxane, Restek). The GC used helium as the carrier gas set at a linear velocity of 33.5 cm/s. The GC oven temperature was held at 35 °C for 3 min, then ramped up to 250 °C at 4 °C/min, and then held at this temperature for 10 min. The interface and MS source temperature was set at 250 °C and 200 °C, respectively, with the MS source operated in electron impact (EI) mode at an ionisation energy of 70 eV.

For aroma analysis, we used peak areas integrating using the main target ion (*m*/*z*) for each compound (Table S1). Compounds were identified by matching their mass spectra to the NIST11 mass spectra library (National Institute of Standards and Technology) and comparison to chemical standards (Table S1). Chromatogram analysis was conducted using GCMSsolutions version 4.20 (Shimazdu, USA).

### 2.3. Sensory Analysis

After five months of bottle aging at 13 °C, sensory analysis was conducted using a sensory panel composed of 17 winemakers from the Willamette Valley, Oregon. The age range of panelists was 25 to 66 and each winemaker had a minimum of 5 years’ experience producing white wine. Panelists were screened for oral lesions, specific anosmia, and cigarette use. A positive response for any of the questions resulted in exclusion. Each panelist tasted 10 wines presented in a random order using an incomplete block design, which included the 8 treatments listed and two randomly designated replicate samples in one two-hour session. Wine glasses were labeled with randomly generated three-digit identifiers. Any background odors were eliminated with air purifiers and the temperature of the room was kept at 20 ± 2 °C.

This experiment utilized Napping^®^ followed by Ultra-flash-profiling (UFP) [23,24]. In brief, sketch paper (50 lb., 45.7 cm × 61 cm) and pens were placed in front of the panelist. Panelists were asked to refrain from smelling the wine samples as mouth feel analysis was the main objective of the sensory tests. They were instructed to immediately take the sample into their mouth. Tasters grouped the wines based on similarity of mouthfeel, with wines placed closer on the paper to wines of similar mouthfeel and wines which were very different in mouthfeel being placed further apart. Once the wines were placed on the paper, each panelist was asked to enrich the wine(s) with descriptors related to mouth feel which would characterize the differences between wines written near the wine/group (UFP). UFP terms were combined when obvious synonyms were utilized by panelists. This study utilized an incomplete block design for replication, where each panelist received two replicate samples per tasting, which resulted in a complete replication of each treatment across all panelists.

### 2.4. Data Analysis

Analysis of variance was used to interpret the chemical parameters with treatment types using R studio version 3.2.1 (R consortium, Boston, MA, USA). Tukey’s HSD test and 95% confidence intervals were utilized to assess the impact of winemaking treatment on alcohol concentration, acetic acid concentration, malic acid degradation, as well as time to complete MLF. Aroma composition was analyzed using principal component analysis. Aroma composition and sensory data analysis was conducted using XLSTAT (Addingsoft co., New York, NY, USA) and the FactoMineR package from R version 3.2.1 [25]. Napping^®^ data were obtained using a tape measure (millimeters) from the left (X) and bottom edges (Y) relative to the original orientation of the paper to the panelists. These measurements were utilized to generate Multiple Factor Analysis. Correspondence analysis was used to evaluate the UFP terms.

## 3. Results

**Figure 2:**
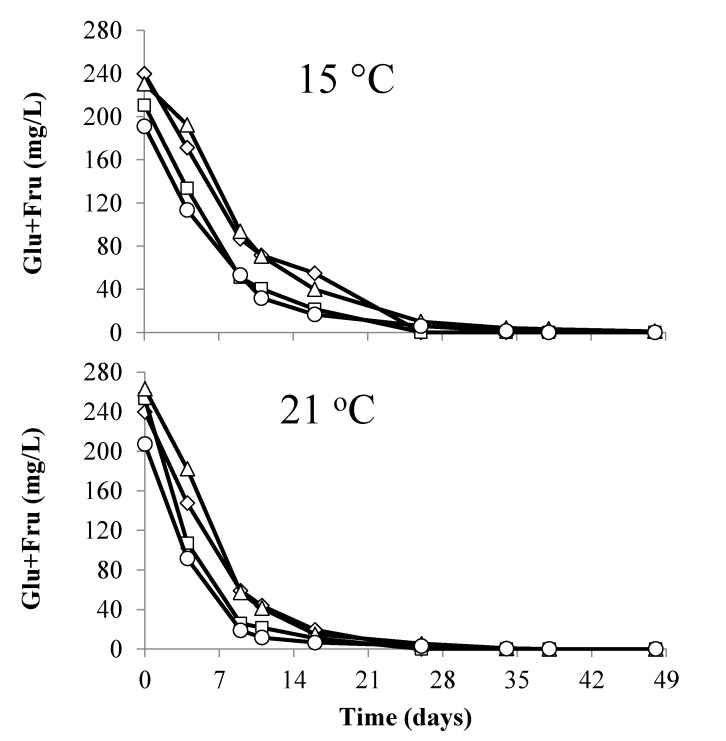
Changes in glucose and fructose during alcoholic fermentation at either 15 or 21 °C. Concurrent AF and MLF conducted by *S. cerevisiae* D47 and *O. oeni* Beta with pre-fermentation addition of *T. delbrukeii* Prelude™ (Δ); AF by *S. cerevisiae* D47 with pre-fermentation addition of *T. delbrukeii* Prelude™ (◊); AF by *S. cerevisiae* D47 (□); concurrent AF and MLF conducted by *S. cerevisiae* D47 and *O. oeni* Beta (◦). Values are means from triplicate fermentations.

**Figure 3:**
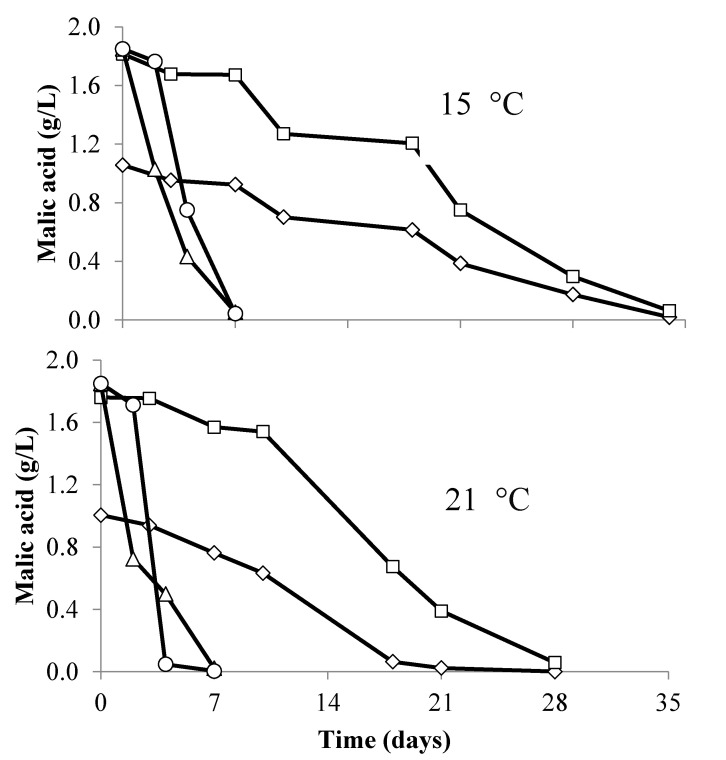
Malic acid concentration during malolactic fermentation conducted at either 15 or 21 °C. Concurrent MLF and AF conducted by *O. oeni* Beta and *S. cerevisiae* D47 with pre-fermentation addition of *T. delbrukeii* Prelude™ (Δ); sequential MLF by *O. oeni* Beta following AF by *S. cerevisiae* D47 with a pre-fermentation addition of *T. delbrukeii* Prelude™ (◊); sequential MLF by *O. oeni* Beta following AF by *S. cerevisiae* D47 (□); concurrent MLF and AF conducted by *O. oeni* Beta and by *S. cerevisiae* D47 (◦). Values are means from triplicate fermentations.

All treatments completed alcoholic fermentation within 35 days, although treatments where *T. delbrueckii* Prelude™ was added were initially slower to metabolize glucose/fructose (Figure 2). MLF completed in 8 days in all concurrently inoculated treatments (malic acid <0.5 mg/L), while it took between four and five weeks to complete in sequential inoculations (Figure 3). When combining the length of time for the completion of both the alcoholic and malolactic fermentation, there were significant differences (*p* < 0.05) between the treatments. Chardonnay wines produced with a concurrent inoculation strategy completed the fermentations in 26 days, while those produced using a sequential fermentation strategy took between 62 and 82 days to complete (Table 1). Sequential fermentations where *T. delbrueckii* had been added pre-AF contained lower concentrations of malic acid at the end of AF than wines where *T. delbrueckii* had not been added (Figure 3). Wines were assessed for a number of parameters after the completion of AF and MLF (Table 1). While there was no significant difference in acetic acid concentration between the fermentation treatments conducted at the same temperature (Table 1), there was a significant difference (*p* < 0.05) in acetic acid between wines fermented at different temperatures. Ferments conducted at 15 °C contained significantly higher concentrations of acetic acid compared to ferments conducted at 21 °C (Table 1).

There were also significant differences (*p* < 0.05) in the final ethanol concentrations of the wines. For fermentations conducted at 15 °C, there were significant differences (*p* < 0.05) between all treatments for ethanol concentration (Table 1). Wines produced by concurrent AF and MLF plus *T. delbruekii* Prelude^TM^ addition pre-fermentation had the lowest ethanol, while ferments conducted by sequential fermentation contained the highest ethanol content. At 21 °C, both the concurrent inoculated wines contained lower alcohol than wines produced by sequential inoculation (Table 1). Overall, the highest ethanol concentration was measured in wines fermented at 15 °C where MLF occurred after alcoholic fermentation, while the lowest was in wines fermented at 21 °C where Prelude™ had been inoculated and MLF occurred concurrently.

**Figure 4:**
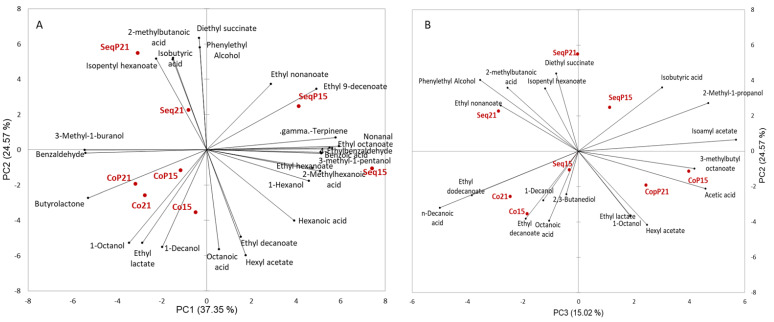
Aroma compound vector loading and means for Sequential (Seq) and Concurrent (Co) treatments at two different temperatures (15 °C and 21 °C) and with inoculation of *T. delbrueckii* (P). (**A**) Contains PC1 and PC2 and (**B**) contains PC2 and PC3.

Principle component analysis of the aroma compounds showed a clear separation between sequentially inoculated wines and concurrent inoculated wines along PC1 and PC2 (Figure 4). A total of 77% of the variance is explained by the first three principal components. Within the sequential inoculations, there was a clear separation by temperature, which was not found in the concurrent inoculated wines.

The influence of *Torulaspora delbrueckii* on aroma composition was seen by wines in the positive F3 direction and wines without *Torulaspora delbrueckii* in the negative F3 direction (Figure 4).

**Figure 5:**
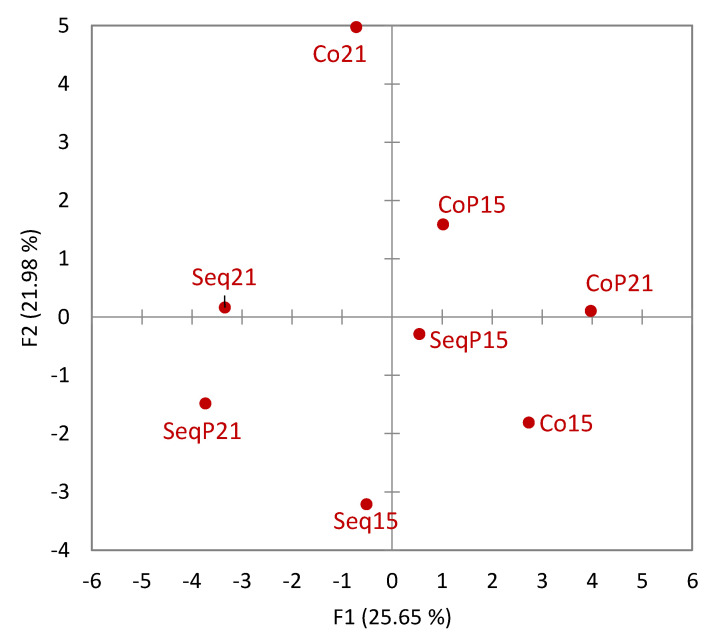
Multiple Factor Analysis of Napping^®^ data for mouthfeel for Sequential (Seq) and Concurrent (Co) treatments at two different temperatures (15 °C and 21 °C) and with inoculation of *T. delbrueckii* (P).

**Figure 6:**
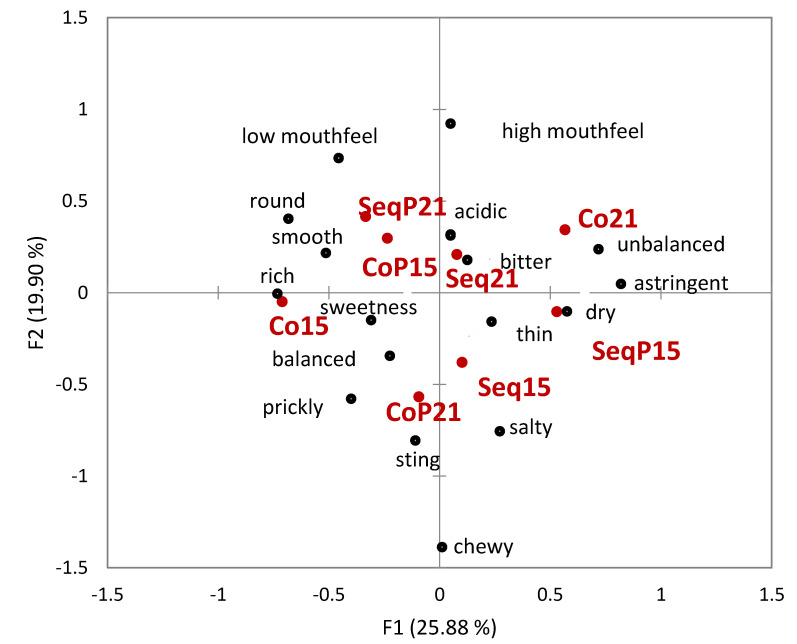
Correspondence analysis of ultra-flash profiling (UFP) descriptors for Sequential (Seq) and Concurrent (Co) treatments at two different temperatures (15 °C and 21 °C) and with inoculation of *T. delbrueckii* (P).

Napping^®^ yielded broadly defined groupings without obvious consistency between temperature or treatment type (Figure 5). While it did appear that wine treatment correlated with differences in mouthfeel, the differences did not appear consistent between temperatures. Correspondence analysis utilizing the UFP data showed how descriptors were associated with each wine (Figure 6). As seen in Figure 6, three of the four co-inoculated treatments are differentiated by groupings on the negative F2 axis; three of the four *T. delbrueckii* treatments also lie on the negative F2 axis. The wines appear to vary in their degree of difference in Napping^®^ location and UFP data.

## 4. Discussion

Concurrent inoculation of AF and MLF during Chardonnay wine production was explored in the present study. While there have been contradictory reports in literature regarding the benefits of this technique regarding fermentation kinetics and sensory impact [26,27,28], the results from the present study support the use of concurrent inoculation as a method to significantly reduce the length of AF and MLF. This is likely due to the choice of yeast and malolactic bacteria used in the present study as others have noted that the specific yeast and bacteria combination can have a significant influence on the success of fermentation [14,29,30,31]. In the present study, the difference between when fermentations were completed in the co-inoculated ferments vs. sequential ferments was as large as 56 days. Concurrent inoculated ferments allow for earlier SO_2_ additions to minimize oxidation and microbial spoilage as well as earlier release of product to the market [15].

The addition of a high population of the non-*Saccharomyces* yeast *T. delbrueckii* did not impact the kinetics of the concurrent inoculated AF and MLF. While interactions between non-*Saccharomyces* yeast and *Saccharomyces cerevisiae* have been reported previously [32], little is known regarding how these yeasts impact *O. oeni*. Results from this study suggest that high populations of *T. delbruekii* will not hinder *O. oeni* conducting MLF, suggesting that concurrent inoculation may still be a viable option in years of high microbial load on grape skins. It would be interesting to perform a concurrent inoculated fermentation where there is a high background population of *H. uvarum* as this yeast is the most common yeast found on grapes at harvest.

Varying the timing of the fermentations (AF and MLF) impacted the aroma composition of the wines, with sequential inoculations also being impacted by fermentation temperature. The choice of yeast and bacteria strains is known to alter aroma composition [12,15] and differences in aroma composition for sequential inoculation compared to concurrent inoculation has also been demonstrated in other wines [12,33]. The main aroma compound differences between the co versus sequential inoculated wines are that the sequentially inoculated wines, specifically at the cooler fermentation temperature, were characterized by a greater number of compounds, specifically ethyl esters. The concurrent inoculated wines were characterized by 1-octanol, 1-decanol, ethyl lactate, and butyrolactone. The sequential inoculations were separated based on temperature, with the higher fermentation temperature (21 °C) wines characterized by isobutyric acid, 2-methylbutanoic acid, diethyl succinate, phenethyl alcohol, and isopentyl hexanoate. The cooler sequentially inoculated wines were characterized by a greater number of aroma compounds including ethyl esters, gamma-terpinene, and 1-hexanol. These results are in agreement with previous work that has shown that sequential and concurrent inoculations alter the fruity and lactic aromas of wines [34]. Ethyl lactate and diethyl succinate are known to influence the buttery and creamy aspects of wine [35], ethyl esters are known to have fruity aromas [36], and fatty acids are linked to both fruity and dairy aromas [37]. These aroma compounds are most likely influencing aroma perception as fatty acids and ethyl esters are known to be important impact odorants [37,38].

Sequential fermentations alter the metabolites available to the malolactic bacteria compared to the yeast, while concurrent inoculated fermentations would have the same available to both. Therefore, the timing of MLF has the potential to change the aroma characteristics of the wine, as described above, which may or may not be desirable depending on the targeted wine style. In addition, wines produced by concurrent inoculated fermentations will be able to have SO_2_ added at an earlier time point and reduce the likelihood of aroma changes due to oxidation. For example, in the present study, no SO_2_ could be added for 60–80 days to wines that underwent sequential fermentations, while SO_2_ could be added after 26 days if wine was produced by concurrent inoculation.

The present study is one of the few to determine mouthfeel differences due to MLF timing and temperature. While mouthfeel differences between the various wines were noted, the differences did not necessarily align with differences in pH, acetic acid, and residual sugar content. Wines also did not group based on temperature of fermentation or timing of the MLF. These findings suggest that while the range of winemaking procedures investigated in this study can affect Chardonnay wine mouthfeel, there was not one dominant factor driving mouthfeel differences.

Although the addition of *T. delbruekii* did not impact fermentation kinetics, it did impact other wine parameters. For example, a drop in malic acid due to the addition of *T. delbrueckii* was noted. Other non-*Saccharomyces* yeasts have the ability to partially degrade malic acid [39,40] and based on our results, *T. delbruekii* also has this trait. *T. delbrueckii* could be a viable alternative to *O. oeni* inoculation for partial degradation of malic acid in cool climates. This is an important consideration for wines with particularly high levels of malic acid, such as sparkling wines, when MLF can be challenging for *O. oeni* to complete. A combination of a non-*Saccharomyces* yeast that can partially degrade the malic acid and *O. oeni* may be helpful in these situations. It could also be a tool for winemakers who do not wish for a complete MLF as *T. delbrueckii* cannot completely utilize the malic acid, but can partially degrade it [41]. A reduction in alcohol was also noted when *T. delbruekii* was added pre-fermentation for the concurrent AF and MLF wines. This reduction in alcohol was not observed if MLF was carried out sequentially. This finding is in alignment with recent studies where an addition of non-*Saccharomyecs* yeast prior to alcoholic fermentation lowered the final alcohol content of the wine [19].

*T. delbrueckii* also altered the aroma composition of the wines. Wines made with this yeast were characterized by diethyl succinate, isobutyric acid, isoamyl acteate, acetic acid, hexyl acetate, and 3-methylbutyl octanoate. The change in aroma composition when *T. delbrueckii* was part of the fermentation was anticipated, as previous work has shown that *T. delbrueckii* alters aroma composition in a similar fashion as shown in our results [42]. The role of *T. delbrueckii* on the mouthfeel perception differences between treatments is not completely understood. Three of the four treatments inoculated with *T. delbrueckii* were consistently grouped together and were all influenced by the descriptive terms balanced, rich, and sweetness. Sequential inoculation and *T. delbrueckii* at 15 °C treatment was grouped separately and was characterized by the terms astringent, unbalanced, thin, and dry. Unlike the concurrent inoculation grouping, this treatment did not have significantly different residual sugar or ethanol, which might explain this discrepancy. Domizio et al. [43] has previously demonstrated the increase in the mannoprotein content of finished wines when *T. delbrueckii* is inoculated. However, mannoprotein differences alone do not appear to account for the differences between treatments of this study. Understanding the metabolism of *T. delbruckii* and the secondary metabolites of malic acid degradation could lead to a new understanding of this yeast’s impact on wine sensory evaluation.

## 5. Conclusions

Concurrent inoculated fermentation of Chardonnay significantly reduced the time needed for the completion of both fermentations. No adverse impacts on wine quality were noted for concurrently inoculated ferments as acetic acid increases were driven by lower fermentation temperature rather than MLF timing. Timing and temperature of MLF impacted volatile aroma composition, while mouthfeel was affected to a lesser extent. The addition of a high population of a non-*Saccharomyces* pre-fermentation did not impact concurrently inoculated MLFs, but did result in significant differences in aroma composition and mouthfeel perception compared to controls. This study has demonstrated the usefulness of concurrent inoculation for a more rapid completion of AF and MLF fermentations as well as how MLF timing and temperature may impact wine qualities such as aroma and mouthfeel.

## Figures and Tables

**Table 1:** Time to completion of alcoholic fermentation (AF) and malolactic fermentation (MLF) of Chardonnay wine treatments by *S. cerevisiae* D47 and *O. oeni* Beta and final wine ethanol and acetic acid concentrations.

Winemaking Treatment	Days to Complete AF & MLF	Alcohol % (*v*/*v*)	Acetic Acid (g/L)
Concurrent AF and MLF at 15 °C	26 ± 0 ^a^	14.14 ± 0.09 ^b^	0.72 ± 0.03 ^a^
Concurrent AF and MLF at 15 °C + pre-ferment addition of *T. delbrueckii*	26 ± 0 ^a^	13.87 ± 0.06 ^a^	0.72 ± 0.03 ^a^
Sequential AF and MLF at 15 °C	68 ± 0 ^b^	14.64 ± 0.06 ^d^	0.70 ± 0.01 ^a^
Sequential AF and MLF at 15 °C + pre-ferment addition of *T. delbrueckii*	82 ± 0 ^c^	14.54 ± 0.08 ^c^	0.71 ± 0.01 ^a^
Concurrent AF and MLF at 21 °C	26 ± 0 ^a^	14.18 ± 0.10 ^b^	0.58 ± 0.01 ^b^
Concurrent AF and MLF at 21 °C + pre-ferment addition of *T. delbrueckii*	26 ± 0 ^a^	13.82 ± 0.04 ^a^	0.59 ± 0.01 ^b^
Sequential AF and MLF at 21 °C	62 ± 0 ^b^	14.55 ± 0.06 ^c^	0.56 ± 0.01 ^b^
Sequential AF and MLF at 21 °C + pre-ferment addition of *T. delbrueckii*	62 ± 0 ^b^	14.43 ± 0.04 ^c^	0.58 ± 0.00 ^b^

^a–d^ Values with different subscripts within each column are significantly different by *p*-value <0.05 according to Tukey’s HSD.

## Supplementary Materials

The following are available online at https://www.mdpi.com/2304-8158/9/6/802/s1, Table S1: Qualification parameters for HS-SPME-GCMS analysis of aroma compounds in Chardonnay wines.
