# Supplementary material for: Impact of the Timing and Temperature of Malolactic Fermentation on the Aroma Composition and Mouthfeel Properties of Chardonnay Wine
Source: Foods. 2020 Jun 18;9(6):802. doi: 10.3390/foods9060802 (PMC7353488; doi:10.3390/foods9060802)
Supplement: Supplementary file 1 [file foods-09-00802-s001.pdf]

Table S1. Qualification parameters for HS-SPME-GCMS analysis of aroma compounds in Chardonnay wines

	**Ret. Time (min)**	**Target ion m/z**	**Confirming ions m/z**	**Purity**	**CAS No.**	**Source**
2-Methyl-1-propanol	6.30	31	55, 47	≥99%	78-83-1	Sigma-Aldrich
Isoamyl acetate	6.73	70	55, 87	≥97%	123-92-2	Sigma-Aldrich
3-Methyl-1-butanol	12.98	55	42, 70	98%	123-51-3	Sigma-Aldrich
γ-Terpinene	13.72			≥95%	99-85-4	Sigma-Aldrich
Ethyl hexanoate	13.74	88	99, 60	≥98%	123-66-0	Sigma-Aldrich
Hexyl acetate	15.79	43	56, 69	≥98%	142-92-7	Sigma-Aldrich
3-Methyl-1-pentanol	18.5			≥99%	589-35-5	Sigma-Aldrich
Ethyl lactate	19.09	45	29, 75	≥98%	97-64-3	Sigma-Aldrich
1-Hexanol	19.59	56	69, 84	98%	111-27-3	Sigma-Aldrich
2-Methylhexanoic acid		60	43, 87	≥99%	4536-23-6	Sigma-Aldrich
Nonanal	20.80	70	41, 82	≥98%	124-19-6	Sigma-Aldrich
Ethyl octanoate	22.46	88	101, 127	≥98%	106-32-1	Sigma-Aldrich
3-methylbutyl hexanoate	23.20	70	99, 117	98%	2198-61-0	TCI America
Acetic acid	23.25	43	60, 15	≥99.5%	64-19-7	Sigma-Aldrich
Benzaldehyde	25.08	77	51, 106	≥98%	100-52-7	Sigma-Aldrich
Ethyl nonanoate	25.78	88	101, 141	≥98%	123-29-5	Sigma-Aldrich
2,3-Butanediol	26.07	45	43, 57	≥99%	513-85-9	Sigma-Aldrich

1-Octanol	26.65	56	70, 84	≥98%	111-87-5	Sigma-Aldrich
Isobutyric acid	27.05	60	87	≥99%	79-31-2	Sigma-Aldrich
γ-Butyrolactone	28.22	42	56, 86	≥99%	96-48-0	Sigma-Aldrich
Ethyl decanoate	28.95	88	101, 155	≥98%	110-38-3	Sigma-Aldrich
3-Methylbutyl octanoate	29.50	127	70, 145	≥98%	2035-99-6	Sigma-Aldrich
2-Methylbutanoic acid	29.96	85	57, 59	≥98%	116-53-0	Sigma-Aldrich
Diethyl succinate	30.08	129	101, 147	≥98%	123-25-1	Acros Organics
Ethyl 9-decenoate	30.43	88	55, 110	≥96%	67233-91-4	Angene International Limited
4-ethylbenzaldehyde	30.63	133	105, 91	98%	4748-78-1	Sigma-Aldrich
1-Decanol	32.57	55	83, 112	≥98%	112-30-1	Alfa Aesar
Ethyl dodecanoate		183	88, 101	≥98%	106-33-2	Sigma-Aldrich
Hexanoic acid	34.78	60	73, 87	≥98%	142-62-1	Sigma-Aldrich
Phenylethyl alcohol	36.207	91	65, 122	≥99%	60-12-8	Sigma-Aldrich
Octanoic acid	40.10	60	73, 101	≥98%	124-07-2	Sigma-Aldrich
Nonanoic acid	42.59	60	115, 129	≥96%	112-05-0	Sigma-Aldrich
Decanoic acid	44.96	60	129, 172	≥99.5%	334-48-5	Sigma-Aldrich
Benzoic acid	48.11	105	179, 135	≥99.5%	65-85-0	Sigma-Aldrich
